# Integrating Multiplex Immunohistochemistry and Machine Learning for Glioma Subtyping and Prognosis Prediction

**DOI:** 10.1002/mco2.70138

**Published:** 2025-04-22

**Authors:** Houshi Xu, Zhen Fan, Shan Jiang, Maoyuan Sun, Huihui Chai, Ruize Zhu, Xiaoyu Liu, Yue Wang, Jiawen Chen, Junji Wei, Ying Mao, Zhifeng Shi

**Affiliations:** ^1^ Department of Neurosurgery Huashan Hospital Shanghai Medical College Fudan University Shanghai China; ^2^ Research Unit of New Technologies of Micro‐Endoscopy Combination in Skull Base Surgery (2018RU008) Chinese Academy of Medical Sciences and Peking Union Medical College Beijing China; ^3^ Department of Neurosurgery Peking Union Medical College Hospital Chinese Academy of Medical Sciences and Peking Union Medical College Beijing China

**Keywords:** glioma, machine learning, mIHC, molecular subtype, prognosis

## Abstract

Glioma subtyping is crucial for treatment decisions, but traditional approaches often fail to capture tumor heterogeneity. This study proposes a novel framework integrating multiplex immunohistochemistry (mIHC) and machine learning for glioma subtyping and prognosis prediction. 185 patient samples from the Huashan hospital cohort were stained using a multi‐label mIHC panel and analyzed with an AI‐based auto‐scanning system to calculate cell ratios and determine the proportion of positive tumor cells for various markers. Patients were divided into two cohorts (training: *N* = 111, testing: *N* = 74), and a machine learning model was then developed and validated for subtype classification and prognosis prediction. The framework identified two distinct glioma subtypes with significant differences in prognosis, clinical characteristics, and molecular profiles. The high‐risk subtype, associated with older age, poorer outcomes, astrocytoma/glioblastoma, higher tumor grades, elevated mesenchymal scores, and an inhibitory immune microenvironment, exhibited IDH wild‐type, 1p19q non‐codeletion, and MGMT promoter unmethylation, suggesting chemotherapy resistance. Conversely, the low‐risk subtype, characterized by younger age, better prognosis, astrocytoma/oligodendroglioma, lower tumor grades, and favorable molecular profiles (IDH mutation, 1p19q codeletion, MGMT promoter methylation), indicated chemotherapy sensitivity. The mIHC‐based framework enables rapid glioma classification, facilitating tailored treatment strategies and accurate prognosis prediction, potentially improving patient management and outcomes.

## Graphical abstract

Our study developed a novel framework for glioma subtyping using multiplexed immunohistochemistry (mIHC) and machine learning. Our study is based on a gating strategy, which utilizes automated image recognition to determine the proportion of tumor cells positive for specific biomarkers within the overall tumor cell population. We refer to this approach as ‘quantitative pathology.’ This mIHC‐based method enables rapid glioma classification, facilitating tailored treatment strategies and more accurate prognosis prediction.

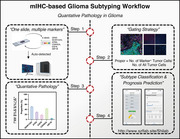

## 1 Introduction

Gliomas, the most prevalent primary malignant brain tumors, pose significant challenges in treatment and prognosis [1]. Even with the standard of care (SOC), patient outcomes remain unsatisfactory, often developing resistance to multiple treatment modalities [1, 2]. Recent research has revealed that gliomas cannot be classified as a single disease entity [3, 4, 5]. Despite similar pathological characteristics between patients or even within different lesions of the same patient, crucial molecular differences may exist, resulting in varied disease progression and treatment responses [6]. The World Health Organization's (WHO) classification guidelines now categorize gliomas based on both pathological and molecular characteristics into IDH (isocitrate dehydrogenase)‐mutant diffuse astrocytomas, IDH‐mutant oligodendrogliomas with 1p19q codeletion, and IDH‐wild‐type glioblastomas (GBM) [7, 8, 9]. Traditional diagnostic methods for gliomas primarily rely on histological and imaging examinations [9]. However, these approaches have limitations in diagnosis, prognostic assessment, and treatment selection. For instance, the morphological characteristics and chromosomal abnormalities of glioma tissues are typically highly heterogeneous, and visual field limitations during pathological reading may lead to interpretation errors in histological examinations. Imaging techniques such as MRI and CT provide information on tumor morphology [9, 10], distribution, and degree of local invasion but offer relatively limited information for evaluating tumor molecular characteristics, prognosis, and treatment response. Therefore, the introduction of molecular detection technology is of great significance in glioma diagnosis. This study aims to provide a more accurate and reliable tumor assessment tool by introducing advanced analytical methods, serving as a strong complement to the existing WHO tumor classification standards.

The prognosis of gliomas is closely related to their molecular characteristics [11]. Hyper‐arrangement and CDKN2A (cyclin‐dependent kinase inhibitor 2A) deletion are associated with increased proliferation of tumor cells [12], reflecting active tumor growth and suggesting poor patient outcomes. Methylation of the MGMT (O6‐Methylguanine‐DNA‐methyltransferase) promoter reduces MGMT expression, making tumor cells dysregulated in the repair mechanism of drug‐induced DNA damage and more sensitive to chemotherapeutic drugs [13]. Consequently, such patients often have a better prognosis. Molecular testing can provide more comprehensive, accurate, and individualized information for glioma diagnosis, further guiding treatment decisions and improving patient treatment outcomes and survival rates.

Glioma diagnosis in current practice relies heavily on imaging and histopathology, which often fail to capture the tumor's extensive molecular heterogeneity [14]. Consequently, clinicians face challenges in accurately predicting prognosis and tailoring treatment strategies. Tumors with similar histopathological features may have distinct genetic profiles, leading to different responses to therapy and varying patient outcomes. Conventional immunohistochemistry (IHC) techniques, often used in glioma pathology, have notable limitations. Each tissue section can only label a single biomarker, limiting the amount of critical prognostic and diagnostic information available from patient samples. Additionally, IHC generally provides only qualitative judgments (positive or negative), with scoring methods like H scores being vulnerable to observer bias [15]. Even with digital analysis, overlapping expression of certain biomarkers—such as PD‐L1 in both tumor cells and M2 macrophages—can interfere with accurate interpretation and ultimately affect clinical diagnosis and treatment decisions [16, 17].

Multiplex immunohistochemistry/immunofluorescence (mIHC/IF) technology allows simultaneous detection of multiple biological markers on a single tissue section [15], enabling parallel monitoring of multiple cells and statistical analysis of cell type, density, spatial relationships, and other information in each tissue region. Furthermore, by “gating” tumor‐specific markers, quantitative analysis of other nonspecific biological markers in specific cell types can be achieved. Combined with an automated end‐to‐end AI rapid pathological diagnosis system, multiple fluorescent protein labeling on the same section through mIHC can produce quantitative statistics and cluster analysis of glioma subtype cell composition.

The glioma immune microenvironment plays a crucial role in tumorigenesis and development [18]. Gliomas with different molecular characteristics have significantly different cell compositions in their immune microenvironments. The accumulation of 2‐HG caused by IDH mutation inhibits the lactate dehydrogenase of CD8+ T cells, thereby reducing glycolysis and disrupting the NAD ± NADH balance, resulting in ROS accumulation and impairing T‐cell proliferation and activity [19]. Additionally, 2‐HG can increase the activity of macrophage tryptophan 2,3‐dioxygenase (TDO) in the tumor microenvironment, allowing l‐tryptophan to be metabolized to the AhR ligand kynurenine (Kyn), activating the AhR signaling pathway, inducing AhR translocation to the nucleus [19], increasing IL‐10 production, leading to decreased macrophage antigen presentation and increased T cell inhibition, and forming an immunosuppressive tumor microenvironment [19]. Moreover, when ATRX mutations are present, radiotherapy and temozolomide chemotherapy can induce high PD‐L1 expression in IDH‐mutant glioma cells, further exacerbating inhibition of the immune microenvironment [20] and promoting tumor immune escape.

In this study, we introduce the concept of “quantitative pathology” and use mIHC technology to quantitatively analyze the composition of tumor cell subtypes in gliomas. We found that the composition of tumor cells in glioma subtypes is significantly different and has guiding significance for prognosis. By constructing a machine learning model, we used mIHC‐based tumor cells' quantitative information to divide gliomas into two groups. These two subtypes of gliomas have significant prognostic differences and distinct molecular, immune, and clinical characteristics. Our study provides a fast and convenient framework for molecular classification of clinical gliomas, which can provide a strong basis for prognosis prediction and accurate treatment of patients.

## 2 Results

### 2.1 The Proportion of Positive Tumor Cells Detected by mIHC Correlates With Mutation Status

**FIGURE 1:**
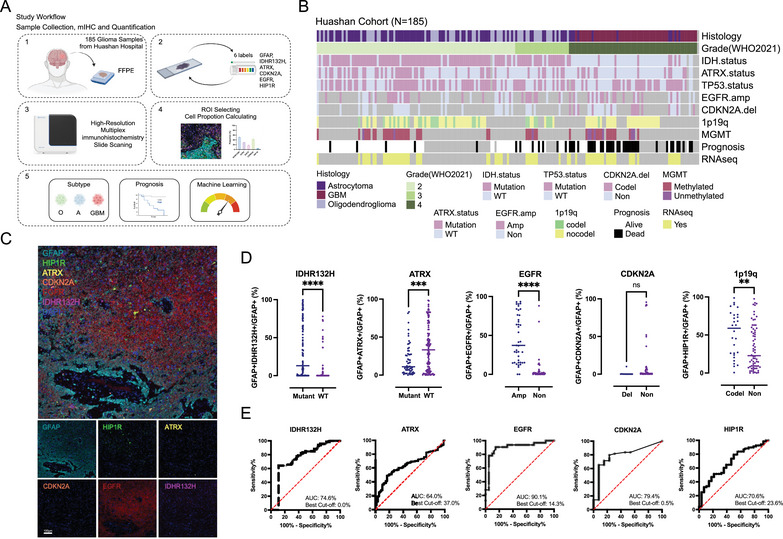
Establishment of a mIHC Panel and correlation between positive tumor cells and mutation status. (A) Study workflow; (B) clinical characteristics of 185 glioma samples; (C) representative figures of mIHC panel; (D) positive tumor cell ratios across different mutation status; (E) receiver operating characteristic (ROC) curves for positive tumor cell ratios across different mutation status. Statistical analyses were conducted using Student's *t*‐test for pairwise comparisons. Significance levels are indicated as follows: *p < 0.05, **p < 0.01, ***p < 0.001, ****p < 0.0001.

To explore the composition of tumor cell subtypes in gliomas, we included paraffin samples from 185 patients diagnosed with gliomas at Huashan Hospital from 2017 to 2020 (see Section 4 for details). The workflow of the patient recruitment process is shown in Figure S1. The patient profile is shown in Table S1. Based on molecular classification indicators of gliomas in previous studies, we simplified these studies: IDHR132H^21^ (R132H mutation in IDH1) was used to detect IDH mutated proteins, ATRX, EGFR (Epidermal Growth Factor Receptor), and CDKN2A were used to reflect ATRX, EGFR, and CDKN2A mutations [12, 22, 23], respectively. HIP1R (huntingtin‐interacting protein 1‐related protein) was used to reflect 1p19q codeletion status [24]. IDHR132H, EGFR, and HIP1R were acquired mutations (gain of functions, GOF), and ATRX and CDKN2A were deletion mutations (loss of functions, LOF). Finally, we selected the following seven indicators as the mIHC panel: IDHR132H, ATRX, EGFR, CDKN2A, HIP1R, GFAP [25] (glial fibrillary acidic protein, marker of glioma cells), and DAPI (4ʹ,6‐diamidino‐2‐phenylindole, marker cell nucleus). Figure 1A,B provides an overview of this study. In brief, we performed mIHC on paraffin sections of 185 glioma patients and obtained the proportion of positive cells in tumor cells through automated high‐throughput mIHC scanning and automatic interpretation. The proportion of positive tumor cells under different grades and pathological classifications was compared. We combined patient prognostic information to explore the impact of various positive cell ratios on prognosis and finally constructed a machine learning model to use cell ratios to predict patient classification and prognosis.

We first explored the relationship between the proportion of various positive cells and mutations (Figure 1C,D). The results showed that the proportion of IDHR132H+ tumor cells in IDH‐mutated gliomas was significantly higher than that in the wild‐type group (Figure 1D), and the proportion of ATRX+ tumor cells in ATRX‐mutated gliomas was significantly lower than that in the wild‐type group (Figure 1D). Regarding EGFR amplification, the proportion of EGFR+ tumor cells in EGFR‐amp gliomas was higher than in EGFR‐wild‐type gliomas. However, in CDKN2A deletion and wild‐type gliomas, the proportion of CDKN2A+ tumor cells showed no significant difference between the two groups. Compared with gliomas without 1p19q codeletion, the proportion of HIP1R+ tumor cells in patients with 1p19q codeletion was significantly higher (Figure 1D). These results demonstrate that the proportion of positive cells calculated by mIHC can indicate the molecular mutation status of gliomas and highlight the molecular heterogeneity in gliomas. Even in glioma patients diagnosed with IDH mutation, wild‐type IDH tumor cells are still present in the tumor tissue. Interestingly, this finding aligns with the conclusions drawn from a recent study utilizing patch‐seq [26].

To further explore the predictive ability of the proportion of positive tumor cells for mutations, we performed ROC analysis on the above indicators. The results showed that the proportion of IDHR132H+ tumor cells had a predictive AUC of 74.6% for IDH mutations, with the best cutoff point at 0.0%, indicating that IDHR132H, as a mutation specifically occurring in tumor cells, has high sensitivity and specificity for mutation status. For ATRX mutation, when the proportion of ATRX+ tumor cells is less than 37.0%, it can be detected as ATRX mutations, with an AUC of 64.0%. ATRX mutation is LOF, and ATRX is also expressed in normal cells (Figure S2). These results suggest that the sensitivity of ATRX mutation detection is affected by the abundance of mutant tumor cells. Similarly, the proportion of EGFR+ tumor cells had a predictive AUC of 90.1% for EGFR amplification, with the best cutoff point at 14.3%, and the proportion of CDKN2A+ tumor cells had a predictive AUC of 79.4% for CDKN2A deletions with the best cutoff point of 0.5%. Gliomas with HIP1R+ tumor cells greater than 23.6% were more likely to carry 1p19q codeletion, with an AUC of 70.6%, suggesting that the proportion of HIP1R+ tumor cells had good predictive power for 1p19q codeletion. These results indicate that the proportion of positive glioma cells detected by mIHC can reflect the molecular mutation status of gliomas. Concurrently, even gliomas diagnosed with certain mutations contain a proportion of wild‐type tumor cells.

### 2.2 Differences in the Proportion of mIHC‐Positive Tumor Cells in Gliomas of Different Grades and Pathological Types and Their Impact on Prognosis

**FIGURE 2:**
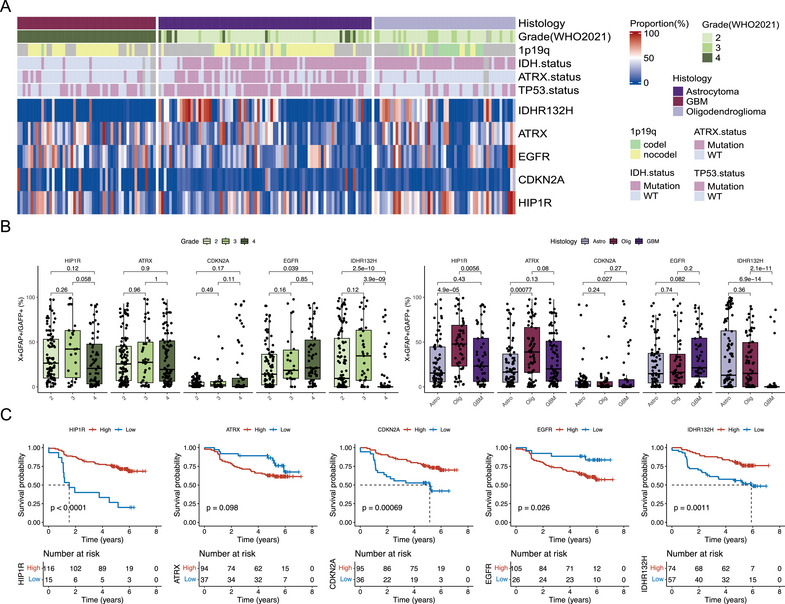
The differences in the proportion of positive tumor cells among different grades and pathological subtypes and their impact on prognosis. (A) Overview of the distribution of mIHC‐based positive tumor cells; (B) positive tumor cell ratios among different grades and pathological subtypes; (C) positive tumor cell ratios’ impact on the patients’ prognosis. Statistical analyses were conducted using ANOVA for multiple group comparisons. A Bonferroni correction was applied to adjust for multiple comparisons.

We then sought to explore the differences in the proportion of positive tumor cells across various grades and pathological subtypes of gliomas (Figure 2A). Initially, we compared the proportion of different positive tumor cells at different grades. The results revealed that compared to high‐grade gliomas, low‐grade gliomas exhibited a significantly increased proportion of HIP1R+ tumor cells, while the proportions of ATRX+ and CDKN2A+ tumor cells showed no significant differences across grades (Figure 2B, left). The proportion of EGFR+ tumor cells was significantly elevated in high‐grade gliomas, suggesting that the proportion of EGFR+ tumor cells could serve as an indicator of disease progression. According to the 2021 WHO CNS guidelines, IDH wild‐type gliomas are classified as Grade 4 GBM [9]. Consistent with previous studies, the proportion of IDHR132H+ tumor cells in Grade 4 gliomas was significantly decreased (Figure 2B, left).

We then compared the proportion of positive tumor cells across different pathological types. Compared to astrocytomas and GBM, oligodendrogliomas exhibited a significantly higher proportion of HIP1R+ tumor cells, which is also associated with the majority of oligodendrogliomas carrying the 1p19q codeletion (Figure 2B, right). Compared to oligodendrogliomas and GBM, astrocytomas showed a significantly lower proportion of ATRX+ tumor cells, which is also consistent with the phenotype of ATRX mutations in astrocytomas [24]. Compared to astrocytomas, GBM demonstrated a significantly decreased proportion of CDKN2A+ tumor cells, indicating an increased proportion of proliferating cells in GBM [27]. Similarly, compared to astrocytomas and oligodendrogliomas, EGFR+ tumor cells also showed an upward trend in GBM (Figure 2B, right). Consistent with previous results [28], the proportion of IDHR132H+ tumor cells in GBM was significantly reduced (Figure 2B, right). These results indicate that the proportion of positive tumor cells differs across grades and pathological types of gliomas, potentially serving as a basis for glioma diagnosis.

Considering the correlation between the proportion of positive tumor cells and the grade and pathological type, we hypothesized that the proportion of positive tumor cells might also be related to the patient's prognosis. We then performed a survival analysis of the proportion of various positive tumor cells (see Section 4 for details). The results showed that patients with a high proportion of HIP1R+, CDKN2A+, and IDHR132H+ tumor cells had a significantly better prognosis than those with a low proportion of positive tumor cells (Figure 2C), while a high proportion of EGFR+ tumor cells was associated with a poor prognosis. The proportion of ATRX+ tumor cells had no significant effect on prognosis (Figure 2C).

**FIGURE 3:**
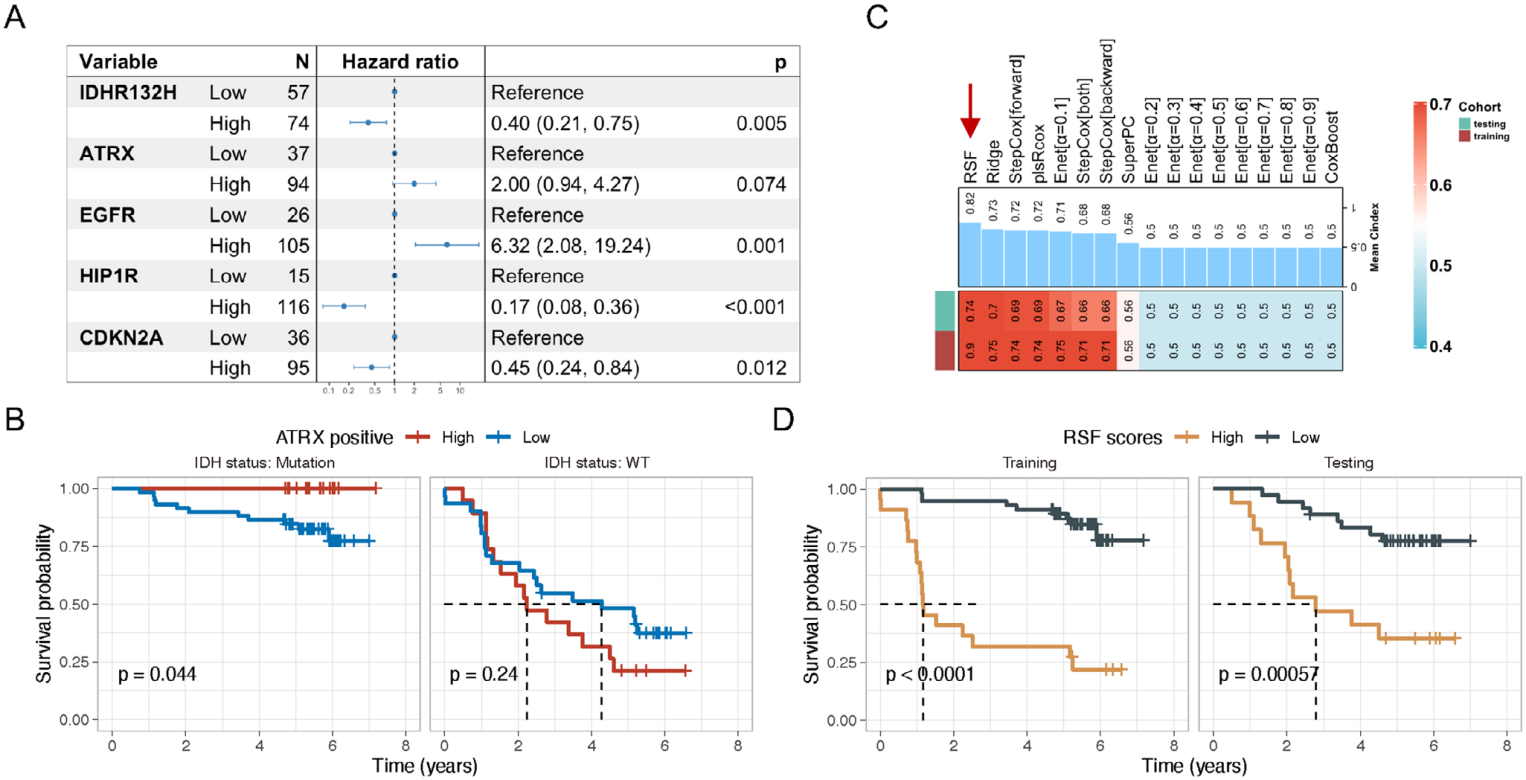
ATRX exhibits significant prognostic differences in different IDH backgrounds and the establishment of a mIHC‐based machine learning model for prognosis prediction. (A) Multivariate Cox regression analysis of positive tumor cells; (B) ATRX exhibits significant prognostic differences in different IDH backgrounds; (C) the mIHC‐based model was developed and validated with a machine learning‐based ensemble program. A total of 17 predictive models were built with the training dataset, and the C‐index of each model in testing sets was further calculated; (D) according to the random survival forest model, the training set and testing set were divided into high‐risk group and low‐risk group respectively.

Recent studies have shown that ATRX mutations have significantly different effects on the biological function and disease prognosis of glioma cells in the context of different IDH mutation statuses. Under IDH wild‐type conditions, ATRX mutation can accelerate tumor growth but simultaneously increase GBM sensitivity to treatment, resulting in a better prognosis for such patients [29]. In the context of IDH mutation, ATRX mutation can cause tumor cells to secrete various cytokine‐mediated inhibitory immune microenvironments and resist treatment [20]. First, we performed survival analysis on ATRX status based on stratified analysis according to IDH mutation status. The results showed that in both IDH mutant and wild‐type conditions, ATRX mutations had no prognostic effect (Figure S3). We then performed a multivariate Cox regression analysis on the proportion of positive tumor cells (Figure 3A). The results showed that the proportion of ATRX+ tumor cells had no significant effect on prognosis (*p* = 0.074). However, stratified analysis according to IDH mutation status found that a low proportion of ATRX+ tumor cells indicates poor prognosis in IDH mutation patients (Figure 3B). These results suggest that it is not that ATRX mutation lacks prognostic significance but that conventional detection methods, such as NGS and IHC, may mistakenly classify some gliomas with ATRX mutation but with a low proportion of mutant tumor cells as ATRX wild‐type. Compared to the ATRX mutation detected by IHC or NGS, the proportion of ATRX+ tumor cells based on mIHC has a more accurate predictive power for patient prognosis.

### 2.3 Construction of a Machine Learning Model Based on mIHC‐Positive Tumor Cell Proportions for Predicting Glioma Patients’ Outcome

The proportions of positive tumor cells from the mIHC panel were then incorporated into 17 machine learning models (see Methods) to predict overall survival. Among these models, the random survival forest (RSF) model demonstrated the best predictive power in both the training dataset and validation set (C‐index: training: 0.90, testing: 0.74), followed by the ridge model (C‐index: training: 0.75, testing: 0.70) and the StepCox [forward] model (C‐index: training: 0.74, testing: 0.69) (Figure 3C). Based on these results, the RSF was selected as the final model. Utilizing the optimal cutoff value of 4.96 derived from the RSF model, patients were stratified into high‐risk and low‐risk groups. In both the training dataset and test set, patients in the high‐risk group exhibited significantly worse prognoses compared to those in the low‐risk group (Figure 3D).

We conducted a comprehensive comparison of clinical characteristics between the high‐ and low‐risk groups across the cohort (Table 1). The results revealed that the mean age of patients in the high‐risk group was 50.6 years, which was significantly higher than the 41.4 years observed in the low‐risk group (*p* < 0.001). In the high‐risk group, the proportion of Grade 4 gliomas was 71.9%, substantially higher than the 16.4% observed in the low‐risk group (*p* < 0.001). Additionally, 61.4% of patients in the high‐risk group were diagnosed with GBM, compared to 13.3% in the low‐risk group. IDH mutation status also differed significantly between the two groups, with 73.7% of patients in the high‐risk group harboring IDH wild‐type tumors, compared to only 19.5% in the low‐risk group (*p* < 0.001). Furthermore, CDKN2A non‐deletion, 1p19q codeletion, and MGMT promoter methylation were more prevalent in the low‐risk group, accounting for 28.1%, 21.1%, and 28.1% of cases, respectively (Table 1). To identify which marker contributes most significantly to the model's predictive accuracy, we conducted a feature importance analysis. Our analysis revealed that HIP1R emerged as the most influential feature, highlighting its potential role in distinguishing glioma subtypes (Figure S4). CDKN2A also demonstrated significant importance, suggesting their relevance in tumor biology and prognosis. HIP1R is associated with the 1p19q codeletion [24], a known favorable prognostic indicator. The deletion of CDKN2A/B has been demonstrated to expedite glioma tumor progression more rapidly than other alterations in the cell cycle pathway [30]. Both CDKN2A gene products, p14 and p16, can inhibit angiogenesis in glioma cells by upregulating tissue inhibitor of metalloproteinase‐3 or downregulating vascular endothelial growth factor, respectively [27, 31], indicating that the loss of CDKN2A may promote blood vessel growth in vivo. Our study found that HIP1R and CDKN2A contribute the most to the model. We hypothesize that high expression of HIP1R and CDKN2A represents the most indolent oligodendrogliomas, which have a favorable prognosis.

**TABLE 1:** Difference of clinical characteristics between high‐risk and low‐risk groups.

	Testing		Training		Overall		*p* value
	High (*N* = 22)	Low (*N* = 52)	High (*N* = 35)	Low (*N* = 76)	High (*N* = 57)	Low (*N* = 128)	
OS.Time (Months)							***< 0.001***
Mean (SD)	40.9 (25.1)	58.5 (16.0)	31.3 (28.7)	62.4 (13.8)	35.5 (27.2)	60.9 (14.8)	
Median [Min, Max]	33.4 [5.92, 78.9]	63.2 [15.9, 84.0]	14.0 [0.0329, 78.9]	65.0 [13.5, 86.1]	25.0 [0.0329, 78.9]	64.4 [13.5, 86.1]	
Missing	5 (22.7%)	16 (30.8%)	13 (37.1%)	20 (26.3%)	18 (31.6%)	36 (28.1%)	
OS							***< 0.001***
Alive	6 (27.3%)	28 (53.8%)	5 (14.3%)	47 (61.8%)	11 (19.3%)	75 (58.6%)	
Dead	11 (50.0%)	8 (15.4%)	17 (48.6%)	9 (11.8%)	28 (49.1%)	17 (13.3%)	
Missing	5 (22.7%)	16 (30.8%)	13 (37.1%)	20 (26.3%)	18 (31.6%)	36 (28.1%)	
Age							***< 0.001***
Mean (SD)	50.6 (13.9)	42.9 (11.6)	50.5 (15.3)	40.3 (11.1)	50.6 (14.6)	41.4 (11.3)	
Median [Min, Max]	50.0 [18.0, 79.0]	40.0 [22.0, 71.0]	50.0 [13.0, 81.0]	38.0 [18.0, 67.0]	50.0 [13.0, 81.0]	39.0 [18.0, 71.0]	
Missing	1 (4.5%)	2 (3.8%)	1 (2.9%)	1 (1.3%)	2 (3.5%)	3 (2.3%)	
Gender							***0.684***
Female	8 (36.4%)	25 (48.1%)	15 (42.9%)	33 (43.4%)	23 (40.4%)	58 (45.3%)	
Male	13 (59.1%)	25 (48.1%)	19 (54.3%)	42 (55.3%)	32 (56.1%)	67 (52.3%)	
Missing	1 (4.5%)	2 (3.8%)	1 (2.9%)	1 (1.3%)	2 (3.5%)	3 (2.3%)	
Grade							***< 0.001***
2	5 (22.7%)	33 (63.5%)	6 (17.1%)	52 (68.4%)	11 (19.3%)	85 (66.4%)	
3	1 (4.5%)	4 (7.7%)	4 (11.4%)	17 (22.4%)	5 (8.8%)	21 (16.4%)	
4	16 (72.7%)	15 (28.8%)	25 (71.4%)	6 (7.9%)	41 (71.9%)	21 (16.4%)	
Missing	0 (0%)	0 (0%)	0 (0%)	1 (1.3%)	0 (0%)	1 (0.8%)	
Histology							***< 0.001***
Astro	6 (27.3%)	26 (50.0%)	10 (28.6%)	38 (50.0%)	16 (28.1%)	64 (50.0%)	
GBM	14 (63.6%)	12 (23.1%)	21 (60.0%)	5 (6.6%)	35 (61.4%)	17 (13.3%)	
Olig	2 (9.1%)	14 (26.9%)	4 (11.4%)	32 (42.1%)	6 (10.5%)	46 (35.9%)	
Missing	0 (0%)	0 (0%)	0 (0%)	1 (1.3%)	0 (0%)	1 (0.8%)	
IDH.status							***< 0.001***
Mutation	5 (22.7%)	35 (67.3%)	8 (22.9%)	65 (85.5%)	13 (22.8%)	100 (78.1%)	
WT	16 (72.7%)	15 (28.8%)	26 (74.3%)	10 (13.2%)	42 (73.7%)	25 (19.5%)	
Missing	1 (4.5%)	2 (3.8%)	1 (2.9%)	1 (1.3%)	2 (3.5%)	3 (2.3%)	
ATRX.status							***0.045***
Mutation	4 (18.2%)	17 (32.7%)	8 (22.9%)	31 (40.8%)	12 (21.1%)	48 (37.5%)	
WT	17 (77.3%)	33 (63.5%)	26 (74.3%)	44 (57.9%)	43 (75.4%)	77 (60.2%)	
Missing	1 (4.5%)	2 (3.8%)	1 (2.9%)	1 (1.3%)	2 (3.5%)	3 (2.3%)	
TP53.status							***1***
Mutation	12 (54.5%)	28 (53.8%)	19 (54.3%)	42 (55.3%)	31 (54.4%)	70 (54.7%)	
WT	9 (40.9%)	22 (42.3%)	15 (42.9%)	32 (42.1%)	24 (42.1%)	54 (42.2%)	
Missing	1 (4.5%)	2 (3.8%)	1 (2.9%)	2 (2.6%)	2 (3.5%)	4 (3.1%)	
EGFR.amp							***0.543***
Amp	4 (18.2%)	12 (23.1%)	6 (17.1%)	18 (23.7%)	10 (17.5%)	30 (23.4%)	
Non	3 (13.6%)	7 (13.5%)	8 (22.9%)	14 (18.4%)	11 (19.3%)	21 (16.4%)	
Missing	15 (68.2%)	33 (63.5%)	21 (60.0%)	44 (57.9%)	36 (63.2%)	77 (60.2%)	
CDKN2A.del							***0.008***
Codel	1 (4.5%)	3 (5.8%)	8 (22.9%)	1 (1.3%)	9 (15.8%)	4 (3.1%)	
Non	7 (31.8%)	12 (23.1%)	6 (17.1%)	24 (31.6%)	13 (22.8%)	36 (28.1%)	
Missing	14 (63.6%)	37 (71.2%)	21 (60.0%)	51 (67.1%)	35 (61.4%)	88 (68.8%)	
1p19q							***< 0.001***
nocodel	11 (50.0%)	20 (38.5%)	13 (37.1%)	24 (31.6%)	24 (42.1%)	44 (34.4%)	
codel	0 (0%)	8 (15.4%)	1 (2.9%)	19 (25.0%)	1 (1.8%)	27 (21.1%)	
Missing	11 (50.0%)	24 (46.2%)	21 (60.0%)	33 (43.4%)	32 (56.1%)	57 (44.5%)	
MGMT							***< 0.001***
Met	4 (18.2%)	11 (21.2%)	2 (5.7%)	25 (32.9%)	6 (10.5%)	36 (28.1%)	
UnMet	3 (13.6%)	3 (5.8%)	2 (5.7%)	1 (1.3%)	5 (8.8%)	4 (3.1%)	
Missing	15 (68.2%)	38 (73.1%)	31 (88.6%)	50 (65.8%)	46 (80.7%)	88 (68.8%)	

*Note*: the bold values represent the information of the compared groups. This formatting is used to clearly distinguish and highlight the specific groups being compared within the table. The italic values, on the other hand, represent the *p*‐value. The inclusion of *p*‐values is to provide a statistical measure that helps in determining the significance of the differences observed between the groups, thereby enabling a more informed interpretation of the data presented.

These findings highlight the distinct clinical and molecular characteristics associated with high‐risk and low‐risk groups as identified by the RSF model, underscoring its potential utility in the prognostic stratification of glioma patients.

### 2.4 RSF Score Is Related to Glioma Malignancy and Molecular Characteristics

**FIGURE 4:**
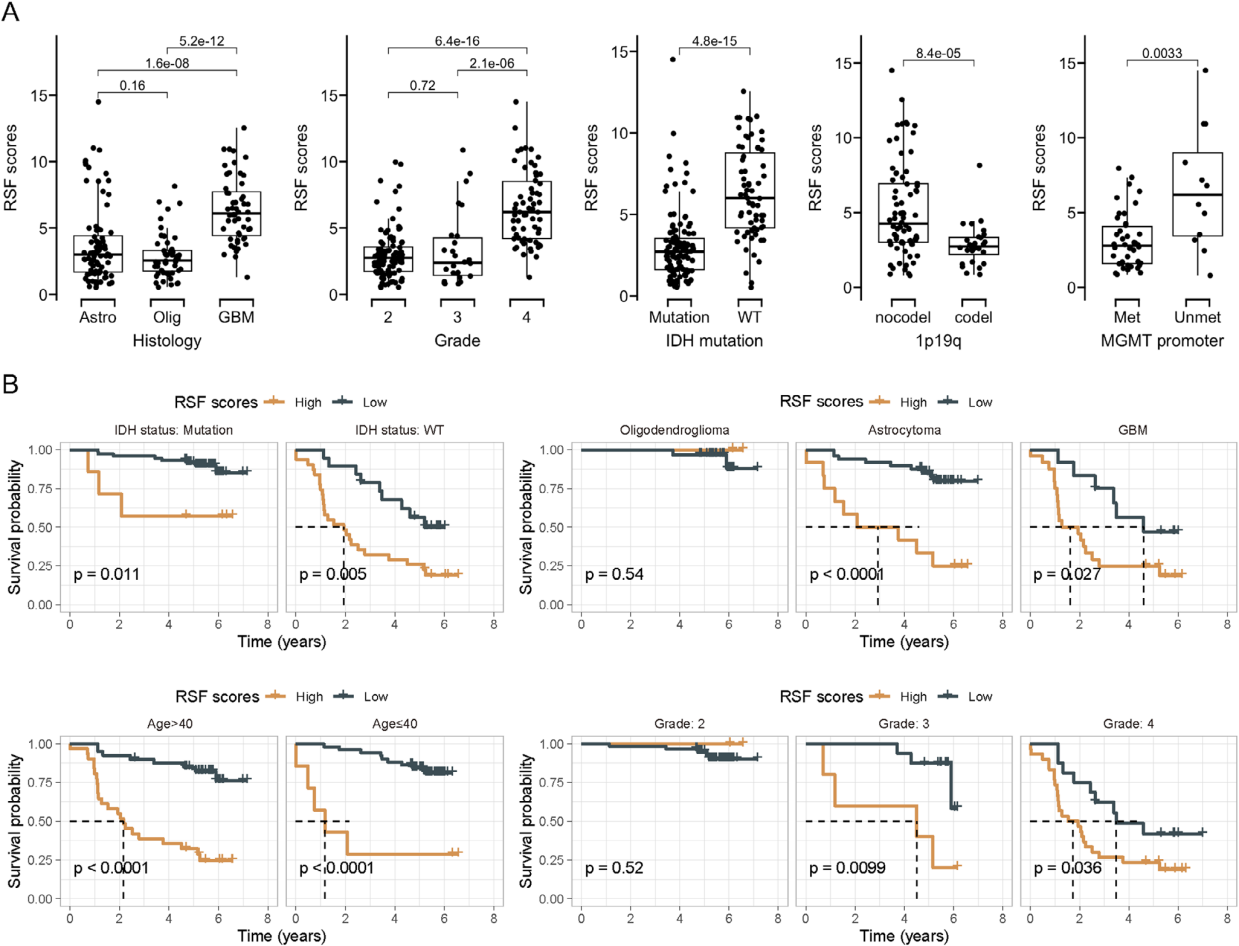
Random survival forest (RSF) scores are related to glioma malignancy and molecular characteristics. Differences in RSF scores in different clinical features (A) histology; grade; IDH mutation; 1p19q; MGMT promoter; (B) Kaplan–Meier survival analysis according to the RSF scores in the context of different IDH mutation status; histology; age group; grade. Statistical analyses were conducted using Student's *t*‐test for pairwise comparisons and ANOVA for multiple group comparisons. A Bonferroni correction was applied to adjust for multiple comparisons.

Next, we investigated the distribution of RSF scores (Figure 4A) across different subtypes, grades, and molecular characteristics. Consistent with our previous findings, GBM exhibited the highest RSF scores, while RSF scores in Grade 4 gliomas were significantly higher than those in Grades 2 and 3, suggesting that RSF scores may serve as an indicator of glioma malignancy. Significant differences in RSF scores were also observed among various molecular types. Gliomas with IDH mutations, 1p19q codeletions, and MGMT promoter methylation demonstrated significantly lower RSF scores compared to their respective control groups, indicating that RSF scores may reflect underlying molecular alterations (Figure 4A).

We further analyzed the prognostic differences between high‐ and low‐risk groups stratified by IDH mutation status, pathological classification, age group, and grade. The results demonstrated that RSF scores maintained good survival prediction ability across different grouping conditions (Figure 4B). For instance, within the IDH mutation group, RSF scores could further identify subgroups with poor prognosis. Similarly, among IDH wild‐type gliomas, patients with lower RSF scores exhibited better prognosis (Figure 4B).

These findings collectively demonstrate that the machine learning‐derived RSF scores correlate significantly with glioma grade, pathological classification, and molecular characteristics. Moreover, patient stratification based on RSF scores consistently identifies two subgroups with markedly different prognoses across various glioma subtypes and molecular profiles. This underscores the potential utility of RSF scores as a robust prognostic tool in glioma management.

### 2.5 High‐Risk Group has a Mesenchymal Phenotype and Shares an Immunosuppressive Microenvironment

**FIGURE 5:**
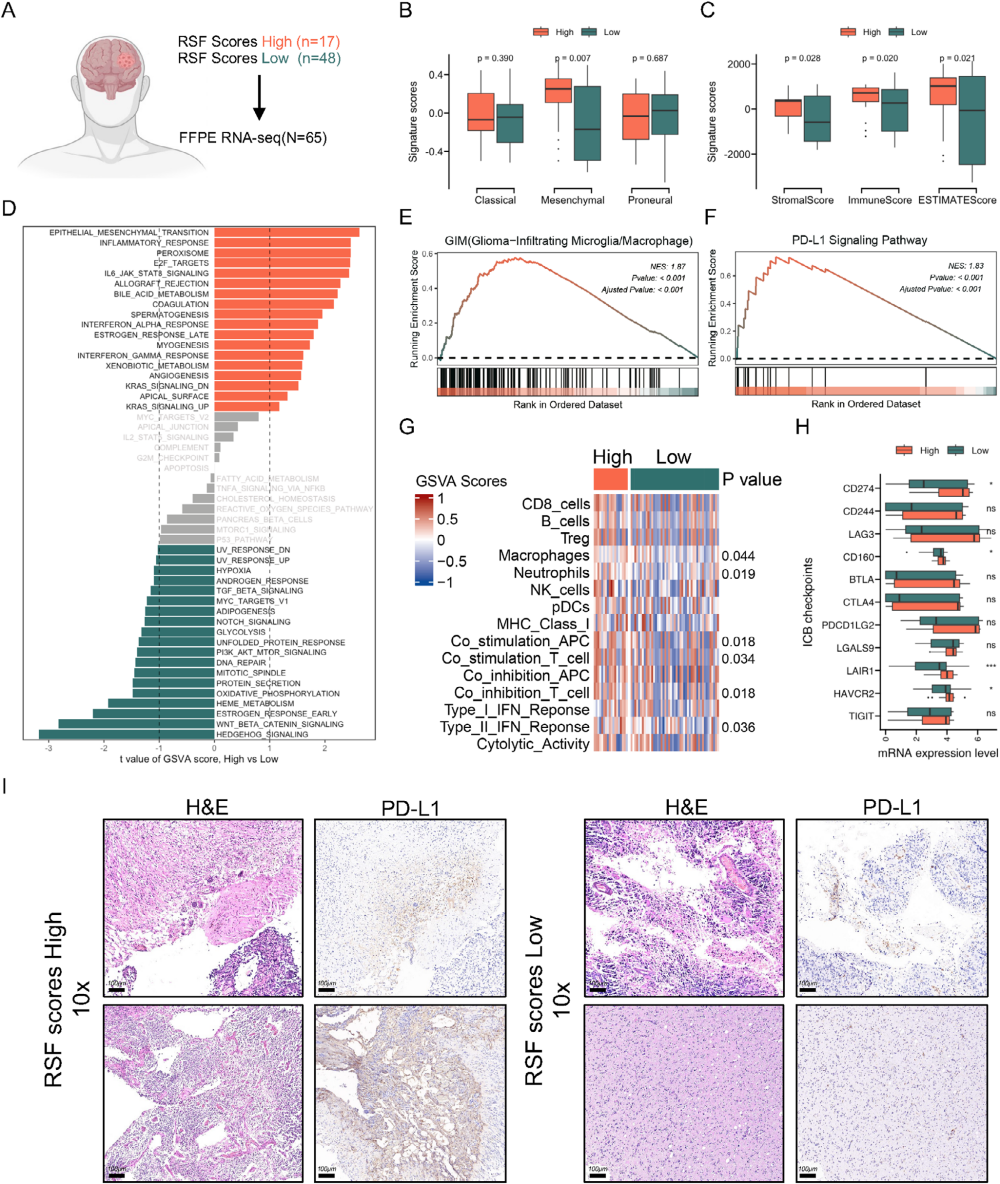
Glioma patients with high RSF scores have an inhibitory immune microenvironment. (A) Study design; (B) difference of classical, mesenchymal, and proneural signature scores between high‐ and low‐risk group; (C) difference of stromal, immune, and ESTIMATE scores between high‐ and low‐risk group; (D) *t* value difference of 50 Hallmark signature scores between high‐ and low‐risk group based on the GSVA algorithm; GSEA of (E) glioma‐infiltrating microglia/macrophages; (F) PD‐L1 signaling pathway between high‐ and low‐risk group; (G) heatmap shows enrichment of immune‐related signatures in two subtypes, the *p* value is labeled on the right side; (H) boxplots show the expression levels of ICB genes across two subgroups; (I) IHC of expression of PD‐L1 protein in high‐ and low‐risk groups. Statistical analyses were conducted using Student's *t*‐test for pairwise comparisons. Significance levels are indicated as follows: *p < 0.05, **p < 0.01, ***p < 0.001, ****p < 0.0001.

Given the close relationship between glioma transcriptional features and patient prognosis [32, 33, 34], we hypothesized that the high and low RSF score groups might exhibit distinct transcriptomic characteristics. To investigate this, we performed RNA sequencing on 65 paraffin samples from our cohort of 185 samples (Figure 5A), comprising 17 patients in the high‐risk group and 48 in the low‐risk group.

We first analyzed the Classical, Mesenchymal, and Proneural signature scores of both groups [35]. Notably, the high‐risk group demonstrated significantly higher mesenchymal scores compared to the low‐risk group (Figure 5B). This finding is particularly relevant as mesenchymal features in glioma typically indicate a high degree of malignancy and poor patient prognosis [35, 36, 37], suggesting that the poor outcomes observed in the high‐risk group may be associated with mesenchymal transformation of glioma. Given the established relationship between the glioma immune microenvironment and molecular subtype [4, 12], we employed the ESTIMATE algorithm [38] to explore differences in stromal and immune scores between the two subgroups. The high‐risk group exhibited significantly higher stromal and immune scores compared to the low‐risk group (Figure 5C). To investigate whether our subtyping method could predict the molecular characteristics of gliomas within both IDH wild‐type and mutant contexts, we stratified the RNA‐seq cohort based on IDH mutation status and subsequently analyzed the molecular features between high‐risk and low‐risk groups within each group. The findings revealed that across both IDH mutant and wild‐type gliomas, the mesenchymal scores of the high‐risk group were significantly elevated compared to those of the low‐risk group. In IDH mutant gliomas, the immune scores of the high‐risk group were markedly higher than that of the low‐risk group, whereas in IDH wild‐type gliomas, the immune scores of the high‐risk and low‐risk groups did not differ significantly (Figure S5). This subtyping method, therefore, demonstrates partial predictive capacity for the molecular features within GBM and IDH‐mutant gliomas. We then utilized the GSVA algorithm to compare differences in Hallmark gene pathways between the high and low‐risk groups. Our results revealed significant upregulation of epithelial–mesenchymal transition, inflammatory response, IL6/JAK/STAT3, coagulation, and IFN‐α response signaling pathways in the high‐risk group (Figure 5D), indicating alterations in immune‐related pathways during disease progression.

GSEA analysis demonstrated significant activation of glioma‐infiltrated macrophages/microglia (GIM) and PD‐L1 signaling pathways in the high‐risk group (Figure 5E,F), suggesting an inhibitory immune microenvironment. Further comparison of immune‐related signaling pathways revealed significant activation of macrophage‐related, neutrophil‐related, co‐stimulation APC, co‐stimulation T cell, co‐inhibition T cell, and Type II IFN response signaling pathways in the high‐risk group (Figure 5G), reinforcing the notion of a highly inhibitory immune microenvironment. Additionally, we examined the expression levels of several inhibitory checkpoint (ICB) genes, given the importance of T cell and natural killer cell inhibition in cancer immune evasion. We observed elevated expression of CD274, CD160, LAIR1 (Leukocyte Associated Immunoglobulin Like Receptor 1), and HAVCR2 (Hepatitis A Virus Cellular Receptor 2) genes in the high‐risk group (Figure 5H). To verify the accuracy of this result, we further performed IHC analysis of PD‐L1 in each of the two patients in the high‐ and low‐risk groups, and the results were consistent with those described above, with patients in the high‐risk group highly expressing PD‐L1 protein (Figure 5I).

To address the potential biases associated with the retrospective collection of our data, we validated our findings using external cohorts from The Cancer Genome Atlas (TCGA). Since TCGA data do not include mIHC validation, we employed a support vector machine (SVM) approach, using our Huashan cohort as reference data (Figure S6A). This method allowed us to perform label transfer and predict the high‐ and low‐risk group labels for each TCGA sample (Figure S6B). First, we analyzed the probabilities of TCGA samples being assigned to high and low‐risk groups at different grades or molecular features. The results showed that the probability of being predicted as a high‐risk group in higher‐grade samples was significantly higher compared to lower‐grade samples. Similarly, the probability of IDH wild‐type gliomas being assigned to the high‐risk group was significantly higher than that of IDH‐mutated gliomas (Figure S6C). The probability of the high‐risk group in MGMT promoter unmethylation gliomas was also significantly higher compared to methylation gliomas (Figure S6C), which is consistent with previous conclusions (Figure 4A), indicating the accuracy of the SVM‐based subtyping of the TCGA cohort. Subsequently, we divided the TCGA cohort into high and low‐risk groups based on the predicted probabilities of being in the high‐risk group, using the optimal cutoff value. We analyzed the impact of high and low‐risk groups on prognosis in different IDH mutation statuses. The results showed that, regardless of whether the gliomas were IDH‐mutated or IDH‐wild‐type, the predicted high‐risk group indicated poor prognosis (Figure S6D). This is consistent with the conclusions obtained in the Huashan cohort (Figure 4B).

Subsequent GSEA and immune checkpoint analyses were conducted on these two groups. The results showed consistent activation of GIM and PD‐L1 signaling pathways in the high‐risk group of the TCGA cohort (Figure S7A,B). Furthermore, the immune‐related signaling pathways, including macrophage‐related, co‐stimulation APC, co‐stimulation T cell, co‐inhibition T cell, and Type II IFN response signaling pathways, were similarly activated in the high‐risk group of the TCGA cohort (Figure S7C). Additionally, the expression levels of ICB genes, such as *CD274*, *CD244*, *LAG3*, *LGALS9*, *LAIR1*, and *HAVCR2*, were also elevated in the high‐risk group of the TCGA cohort (Figure S7D). These findings confirm the reliability of our conclusions and provide further support for the inhibitory immune microenvironment characterized in the high‐risk group.

Collectively, these findings suggest that the high‐risk group identified by our model is characterized by a mesenchymal phenotype and a highly suppressed immune microenvironment. This may represent a biological mechanism underlying the poor outcomes observed in patients classified as high‐risk and offers potential avenues for targeted therapeutic interventions.

## 3 Discussion

**FIGURE 6:**
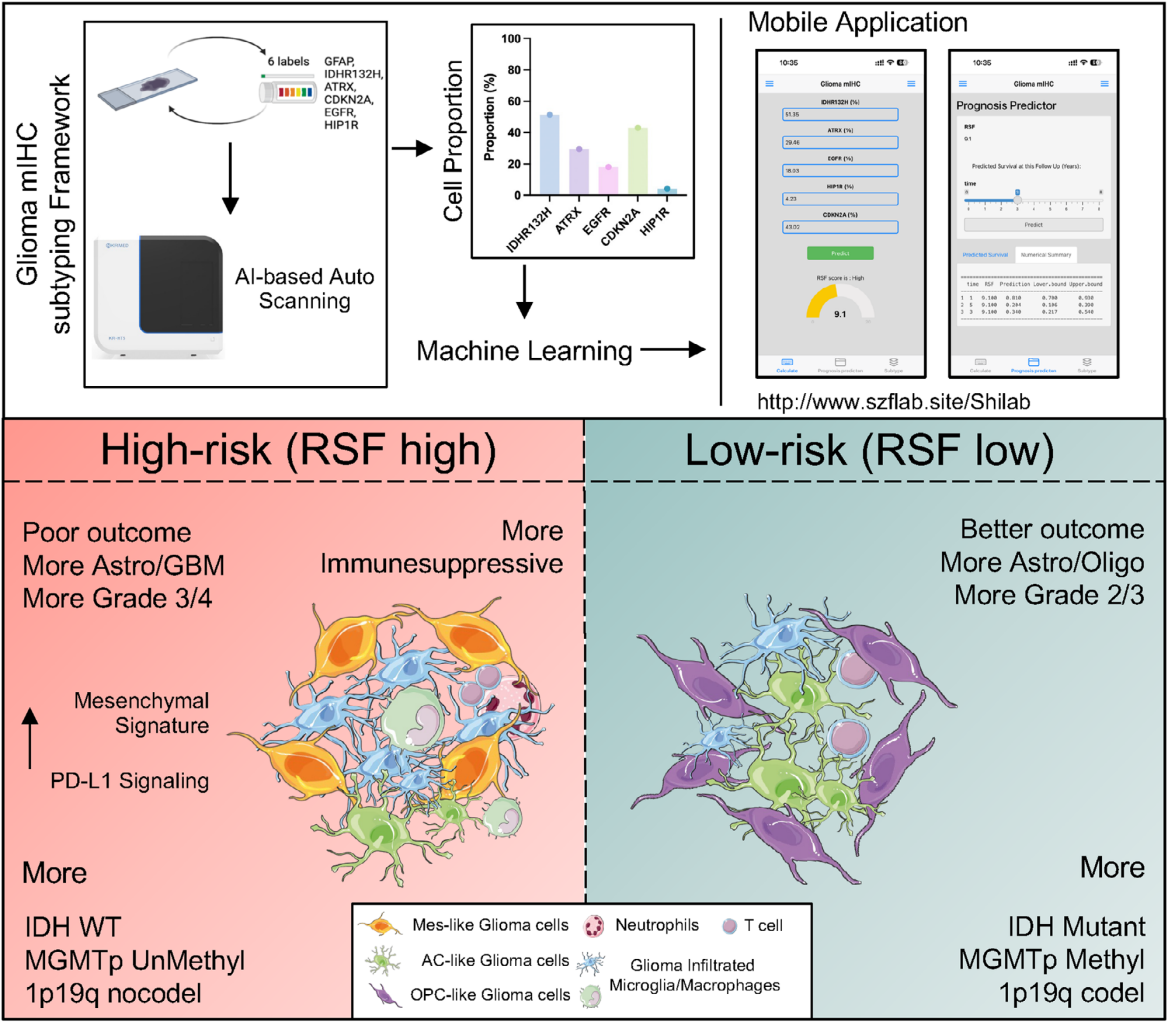
Construction of the mIHC‐based subtyping framework and overview of the two glioma subtypes.

In this study, we established a novel framework for glioma subtyping based on mIHC (Figure 6). The pipeline is as follows: After selecting intraoperative or paraffin‐embedded sample sections, we applied an mIHC panel for fluorescent labeling. An AI‐based auto‐scanning system was then used to automatically read the mIHC slides and calculate cell ratios, yielding the proportion of positive tumor cells for various markers. We then developed a machine learning model and built a mobile phone application based on this model (Figure 6). By inputting the proportions of various positive tumor cells into the mobile application (http://szflab.site/Shilab), we can rapidly obtain a risk score and classify the patient's subtype.

These two subtypes demonstrate significant differences in prognosis, clinical characteristics, and molecular biology. Compared to the low‐risk group, the high‐risk group typically comprises older patients with poorer prognosis, is pathologically inclined towards astrocytoma or glioblastoma multiforme (GBM), and is predominantly of higher grade (Grade 3 or 4). Additionally, these patients usually exhibit higher mesenchymal scores and an inhibitory immune microenvironment. Molecularly, they tend to be IDH wild type, lack 1p19q codeletion, and show MGMT promoter unmethylation (Figure 6), suggesting potential resistance to chemotherapy and necessitating close follow‐up. Conversely, the low‐risk group typically consists of younger patients with better prognosis. Pathologically, these tumors are more likely to be astrocytomas or oligodendrogliomas, predominantly of lower grade (Grade 2 or 3). Molecularly, these patients often harbor IDH mutations, 1p19q codeletion, and MGMT promoter methylation (Figure 6), indicating potential sensitivity to chemotherapy.

Glioma was one of the first cancers to incorporate molecular typing into diagnosis [7, 39]. Molecular typing is closely related to patient prognosis and can guide treatment strategies to some extent [40]. For instance, patients with IDH mutations have significantly better prognosis than those with IDH wild‐type. Patients with MGMT promoter methylation have reduced MGMT expression, implying diminished DNA repair capacity and an increased likelihood of benefiting from chemotherapy [41]. Current clinical practice often uses binary classifications (e.g., WT or mutant, 1 or 0, + or −) as the basis for molecular typing. However, most gliomas exhibit somatic mutations, and even in gliomas diagnosed with IDH mutations, not all tumor cells are IDH‐mutated. Moreover, noncancerous components of tumor tissue, such as infiltrating stromal and immune cells, dilute tumor purity and may confound genomic mutation profiling and pathological biomarker identification [42, 43]. It is suggested that tumor purity above 60% better meets the requirements for mutational invocation [43, 44], and the impact of tumor purity on genomic mutation profiling and pathological analysis should be fully considered [44].

Few studies have explored the relationship between the proportion of mutant tumor cells and clinical molecular typing. Our study, which included 185 glioma patients, investigated this relationship using the proportion of positive tumor cells obtained by mIHC. We found a significant correlation between the proportion of positive tumor cells and mutation status and observed that the proportion of mutant tumor cells differed among patients with the same type of glioma diagnosed as mutant. Notably, we found that using the proportion of mutation‐positive cells as a variable was more accurate in predicting patient prognosis than binary mutation classification (Figures 3B and S3). Compared to conventional “qualitative” mutation pathology information, we defined the proportion of mutant cells obtained by mIHC as “quantitative pathology.”

During glioma development, the intrinsic biological changes of tumor cells are in constant exchange with the surrounding tumor microenvironment [45]. This interaction occurs not only through direct cell‐to‐cell communication but also via chemokines, cytokines, or extracellular vesicles. This interplay promotes glioma progression and invasion, mediates tumor cell immune evasion, and contributes to treatment resistance. The intrinsic molecular characteristics of gliomas are closely related to the composition of their microenvironment [40, 46]. Wild‐type and mutant IDH gliomas have distinct immune microenvironments [47, 48, 49]. The former has more tumor‐associated lymphocytes and myeloid cells and highly expresses PD‐L1. In IDH‐mutant gliomas, the mutant IDHR132H produces 2‐HG, which accumulates and inhibits the lactate dehydrogenase of CD8+ T cells, thereby reducing glycolysis and disrupting the NAD+/NADH balance. This leads to ROS accumulation, impairing T cell proliferation and activity. The (inactive) mutation of ATRX disrupts the gene regulation of glioma chromatin remodeling, promoting the stabilization of glioma cells. In cell line experiments, radiotherapy and temozolomide chemotherapy‐induced high expression of PD‐L1 in IDH‐mutant glioma cells with ATRX mutations, promoting tumor immune escape [20]. During disease progression, the intrinsic characteristics of gliomas change dynamically. Studies have shown that gliomas increase resistance to treatment through mesenchymal transformation, which is accompanied by interaction with tumor‐associated myeloid cells and mediates the generation of an inhibitory immune microenvironment [12, 50].

In this study, we constructed an mIHC‐based subtyping system through machine learning. The high‐risk group demonstrated higher mesenchymal‐like signature scores, suggesting increased malignancy. Additionally, the high‐risk group showed higher levels of immune and stromal scores and increased infiltration of myeloid cells. Several ICB genes were also highly expressed in high‐risk groups, including *CD274*, *CD160*, *LAIR1*, and *HAVCR2*. These findings suggest that the tumor microenvironment of the high‐risk group is highly immunosuppressive, and immunotherapy may be effective in this type of glioma. This indicates that our subtyping system, based on the proportion of intrinsically positive tumor cells in gliomas, may provide further insight into the composition of the tumor microenvironment, offering a new basis for therapeutic modality selection. While our study focuses on computational predictions based on mIHC data, future work will incorporate functional validation experiments to confirm the biological differences observed between high‐ and low‐risk glioma subtypes. In vitro experiments using tumor organoid models, as well as in vivo mouse studies, will be conducted to assess how these subtypes respond to treatment, further validating the clinical relevance of our classification framework.

In addition to mIHC, emerging technologies such as single‐cell RNA sequencing and transcriptomic analysis offer unprecedented resolution in understanding tumor heterogeneity. The combination of single‐cell and transcriptomic sequencing offers a powerful framework for understanding glioma heterogeneity [51, 52], revealing how specific cell populations contribute to the overall transcriptomic landscape and aiding in the identification of therapeutic mechanisms and resistance pathways. These methods can identify distinct cellular subpopulations within gliomas, further elucidating how genetic and molecular variations contribute to treatment resistance and disease progression.

The mIHC‐based framework is designed for integration into existing clinical pathology workflows. Given that most large hospitals are equipped with IHC facilities, the transition to mIHC would require only modest infrastructure upgrades. Furthermore, the AI‐based model ensures rapid, automated analysis, reducing time and labor costs. In resource‐limited settings, where full adoption of the mIHC panel may be challenging, a simplified version focusing on key markers could be developed. This could provide significant clinical benefit while remaining cost‐effective. A key limitation of our study is that the patient cohort was sourced from a single center, which may limit the generalizability of our findings. Future work will focus on validating our mIHC‐based classification system in larger, multicenter cohorts. This will ensure that the model can be applied across diverse populations, including those from different geographic and ethnic backgrounds. Also, the integration of AI into clinical decision‐making is an exciting frontier in modern healthcare [53]. AI models such as the one developed in this study can assist clinicians by providing real‐time prognostic information based on complex molecular data. Additionally, large‐scale AI models, such as ChatGPT [54], have the potential to aid in clinical decision‐making by quickly synthesizing vast amounts of research data and patient information.

Incorporating the latest developments in clinical trials for glioma treatment is crucial for highlighting the clinical translational potential of our study. Recent advancements have focused on innovative therapeutic strategies, including targeted therapies, immunotherapy, and combinatorial approaches that enhance treatment efficacy [14]. These trials provide valuable insights into patient stratification and personalized medicine, underscoring the importance of molecular profiling in treatment selection. As our study emphasizes the significance of specific mIHC markers in glioma subtyping and prognosis, integrating these findings with ongoing clinical trials could lead to more effective and tailored treatment protocols for glioma patients.

In conclusion, our study constructs a novel framework for mIHC‐based glioma subtyping and prognosis prediction. Compared to traditional IHC methods, our approach not only reduces subjectivity and improves diagnostic consistency but also provides more accurate data support for the WHO tumor classification standards. Additionally, by quantitatively analyzing positive signal cells, our method can more precisely evaluate tumor characteristics, offering significant advantages for clinical applications. By combining machine learning methods, we identified two glioma subtypes based on the proportion of mIHC‐positive tumor cells. This classification system demonstrates good predictive power across various clinical contexts. Moreover, the two glioma subtypes exhibit significantly different microenvironment compositions, potentially providing new therapeutic targets for immunotherapy. Further investigation of this mIHC‐based subtyping framework in larger patient cohorts is necessary to determine its potential as a clinically applicable biomarker.

## 4 Materials and Methods

### 4.1 Study Population

This study collected tumor paraffin tissues from 185 glioma patients in Huashan Hospital between 2017 and 2021. The clinical characteristics of the patients are shown in Table S1. Of the 185 samples, 65 received RNA‐seq. This study was conducted according to the Declaration of Helsinki (as revised in 2013) and approved by the Ethics Committee of Huashan Hospital, Fudan University. All patients provided written informed consent before enrollment in this study.

### 4.2 mIHC Staining and Quantification

To identify the proportion of mutant tumor cells in gliomas, we performed mIHC staining according to the manufacturer's protocol using the KuoRan 7‐plex IHC Kit (https://www.krgene.com/services/ngpranseshijihe/, KuoRan, Shanghai, China). Antibodies include anti‐IDHR132H (ZM‐0447, ZSGB‐BIO, China), anti‐ATRX (ab97508, Abcam, USA), anti‐EGFR (ZA‐0505, ZSGB‐BIO, China), anti‐CDKN2A (ab270058, Abcam, USA), anti‐HIP1R (ab140608, Abcam, USA), anti‐GFAP (ab7260, Abcam, USA), and DAPI (ab285390, Abcam, USA). In short, paraffin section slides are dewaxed and rehydrated. Antigen repair is then performed by microwave as follows: place the slides in 100 mL of antigen repair solution (citric acid solution), boil, cool, wash with distilled water, then transfer the slides to a 1X TBST jar containing the slides (TBS [pH 7.4] plus 0.1% Tween 20). Next, cover the sections with the blocking solution and incubate for 10 min, then, add the primary antibody and incubate at room temperature, followed by horseradish peroxidase‐conjugated secondary antibody and teramide signal amplification (TSA). Stain the sequential antibodies by repeating the procedure as described above. After labeling all antigens, stain the nuclei with DAPI. Finally, mIHC slides were scanned using the KR‐HT5 tissue section quantitative analysis system (KuoRan, Shanghai, China) and quantified on the KRIAS professional quantitative pathology analysis software (KuoRan, Shanghai, China). We defined GFAP‐positive cells as tumor cells and calculated the proportion of mutation‐positive tumor cells by calculating the proportion of positive cells in tumor cells.

### 4.3 Immunohistochemistry

For this study, IHC staining was performed using a commercial kit (Servicebio, #G1313‐100T), following the manufacturer's protocol. The process involved several key steps: (1) deparaffinization and Rehydration: sections underwent sequential immersion in deparaffinization solutions I, II, and III (Servicebio, #G1128‐500ML) for 10 min each, followed by three 5‐min immersions in absolute ethanol. The sections were then rinsed with distilled water. (2) Antigen retrieval: sections were immersed in 1X citrate buffer (Servicebio, #G1219‐1L) and heated to boiling. The temperature was maintained above 98°C for 15–20 min, after which the sections were allowed to cool naturally to room temperature. (3) Endogenous peroxidase blocking: sections were treated with 3% hydrogen peroxide at room temperature for 10 min. This was followed by three 5‐min washes in PBS using a decolorizing shaker. Protein blocking: a 3% BSA working solution was applied to completely cover the tissue, followed by incubation at room temperature for 30 min. (4) Antibody incubation: primary antibody (PD‐L1, ab205921, Abcam, USA) working solution (1 µg/mL) was applied to fully cover the tissue and incubated overnight at 4°C. Subsequently, sections were incubated with the secondary antibody (IgG H&L [HRP], ab205718, Abcam) working solution (1/10,000). (5) To detect tissue‐bound immune complexes, sections were incubated with the peroxidase substrate diaminobenzidine (DAB) at room temperature for 5–10 min.

### 4.4 Bioinformatics Analysis

ESTIMATE was used to evaluate the immune cell and stromal scores of each sample [38]. Gene Set Enrichment Analysis was performed on the differential genes of the two groups of samples using the R package “clusterProfiler” [55], and single‐sample gene set enrichment analysis (ssGSEA) was performed with the R package “GSVA” to quantify the enrichment level of the characteristic gene set [56].

### 4.5 Processing of RNA‐Seq Reads

RNA‐seq reads were mapped to the genome, and genes were annotated as previously described. Briefly, FASTQ files were assessed with FASTQC v0.12.1 (https://www.bioinformatics.babraham.ac.uk/projects/fastqc/, https://github.com/s‐andrews/FastQC). Using trim_galore (https://github.com/FelixKrueger/TrimGalore) to remove adapters and filter out low‐quality reads with parameters: trim_galore ‐q 25 –phred33 –length 36 –stringency 3 –paired. Using hisat2 [57] for sequence alignment, H. sapiens_grch38_genome tar.gz is used as a reference genome file (https://daehwankimlab.github.io/hisat2/download/). Gencode.v44.annotation.gtf.gz is used as the reference genome annotation file (https://www.gencodegenes.org/human/). Mapped output BAM files were analyzed with featureCounts [58] to estimate gene abundance and obtain count reads overlapping a gene with parameters: featureCounts ‐T 10 ‐p ‐t exon ‐g gene_id ‐a gencode.v44.annotation.gtf ‐o *.bam.

### 4.6 Statistical Analysis

We applied the survival (V3.5‐8, https://cran.r‐project.org/web/packages/survival/) and survminer (V0.4.9, https://cran.r‐project.org/web/packages/survminer) R packages to assess the correlation between the proportion of mutation‐positive tumor cells and overall survival. We calculated the optimal cutoff value using the surv_cutpoint function, divided the patients into high and low groups, calculated the differences in the group using the log‐rank test, and implemented the Kaplan–Meier method in the ggsurvplot function to plot the survival curve. In the machine learning part, we used 17 machine learning algorithms, including RSF and ridge regression. We randomly grouped 185 samples according to the ratio of the training set to the testing set, 0.6:0.4. The machine learning model was used to predict the prognosis of the training set and the testing set. C‐index was used to evaluate the accuracy of the model. Statistical differences between multiple groups of independent samples were calculated using the Kruskal–Wallis rank sum test (V3.6.0), and the Wilcoxon rank sum test was used for comparison between the two groups; the multi‐hypothesis test used Bonferroni, Benjamini–Hochberg, or Holm methods to adjust the *p* value. All statistical tests are completed based on GraphPad 8 and R software (v4.2.0). In this study, *p* < 0.05 was defined as statistically significant.

## Author Contributions

Z.S., Y.M., and J.W. ideated and managed the work presented in this paper. Z.S. and Z.F. also performed experimental design. H.X., Z.F., S.J., and M.S. wrote the manuscript and prepared all the figures/tables. H.C., R.Z., Z.F., J.C., and Y.W. performed tumor sample collection and patients’ follow‐up. All authors approve the final version of the manuscript.

## Ethics Statement

This study was approved by the Ethics Committee of Huashan Hospital, Fudan University (2023‐1012‐HS). All patients provided written informed consent before enrollment in this study.

## Conflicts of Interest

The authors declare no conflicts of interest.

## Supporting information



Supporting Information

## Data Availability

The processed transcriptomics data can be obtained from the Synapse database (https://www.synapse.org/) under Accession Number syn61892120. All processed data and R codes used in this study can be obtained from the corresponding author upon reasonable request.
