# Supplementary material for: Integrating Multiplex Immunohistochemistry and Machine Learning for Glioma Subtyping and Prognosis Prediction
Source: MedComm (2020). 2025 Apr 22;6(5):e70138. doi: 10.1002/mco2.70138 (PMC12013734; doi:10.1002/mco2.70138)

Supplementary Materials

Integrating Multiplex Immunohistochemistry and Machine Learning for Glioma Subtyping and Prognosis Prediction

Houshi Xu[1,2,3#], Zhen Fan[1,2#], Shan Jiang[1,2#], Maoyuan Sun[1,2#], Huihui Chai[1,2], Ruize Zhu[1,2], Xiaoyu Liu[3], Yue Wang[1,2,3], Jiawen Chen[1,2], Junji Wei[2,3]*, Ying Mao[1,2]*, Zhifeng Shi[1,2]*

[1]Department of Neurosurgery, Huashan Hospital, Shanghai Medical College, Fudan University, Shanghai, China

[2]Research Unit of New Technologies of Micro-Endoscopy Combination in Skull Base Surgery (2018RU008), Chinese Academy of Medical Sciences and Peking Union Medical College (CAMS & PUMC), Beijing, China.

[3]Department of Neurosurgery, Peking Union Medical College Hospital, Chinese Academy of Medical Sciences and Peking Union Medical College (CAMS & PUMC), Beijing, China

[#]These authors contributed equally to this work

*Correspondence:

Junji Wei, E-mail: weijunji@pumch.cn

Ying Mao, E-mail: maoying@fudan.edu.cn

Zhifeng Shi, E-mail: shizhifeng@fudan.edu.cn

Table S1: Clinical characteristics of 185 patients in this study.

Figure S1: Workflow of patient recruitment process

Figure S2: Expression of ATRX in cells of glioma tissue.

Figure S3: ATRX mutation status' effect on prognosis in IDH mutant or wild-type gliomas.

Figure S4: Feature importance of the RSF model.

Figure S5: Comparison of molecular characteristics between high-risk and low-risk glioma subtypes within IDH wild-type and IDH-mutant contexts.

Figure S6: Validation of mIHC-defined subgroups in TCGA-GBMLGG cohort.

Figure S7: Immune characteristics of SVM-based predicted subgroups of TCGA GBMLGG cohort.

Supplementary Table 1: Clinical characteristics of 185 patients in this study.

	All (N=185)
**Time(Months)**	
Mean (SD)	53.3 (22.5)
Median [Min, Max]	62.4 [0.0329, 86.1]
Missing	54 (29.2%)
**OS**	
Alive	86 (46.5%)
Dead	45 (24.3%)
Missing	54 (29.2%)
**Age**	
Mean (SD)	44.2 (13.1)
Median [Min, Max]	42.5 [13.0, 81.0]
Missing	5 (2.7%)
**Gender**	
Female	81 (43.8%)
Male	99 (53.5%)
Missing	5 (2.7%)
**Grade**	
2	96 (51.9%)
3	26 (14.1%)
4	62 (33.5%)
Missing	1 (0.5%)
**Histology**	
Astro	80 (43.2%)
GBM	52 (28.1%)
Olig	52 (28.1%)
Missing	1 (0.5%)
**IDH.status**	
Mutation	113 (61.1%)
WT	67 (36.2%)
Missing	5 (2.7%)
**ATRX.status**	
Mutation	60 (32.4%)
WT	120 (64.9%)
Missing	5 (2.7%)
**TP53.status**	
Mutation	101 (54.6%)
WT	78 (42.2%)
Missing	6 (3.2%)
**EGFR.amp**	
Amp	40 (21.6%)
Non	32 (17.3%)
Missing	113 (61.1%)
**CDKN2A.del**	
Codel	13 (7.0%)
Non	49 (26.5%)
Missing	123 (66.5%)
**1p19q**	
codel	28 (15.1%)
nocodel	68 (36.8%)
Missing	89 (48.1%)
**MGMT**	
Met	42 (22.7%)
UnMet	9 (4.9%)
Missing	134 (72.4%)

Supplementary Figure 1: Workflow of patient recruitment process

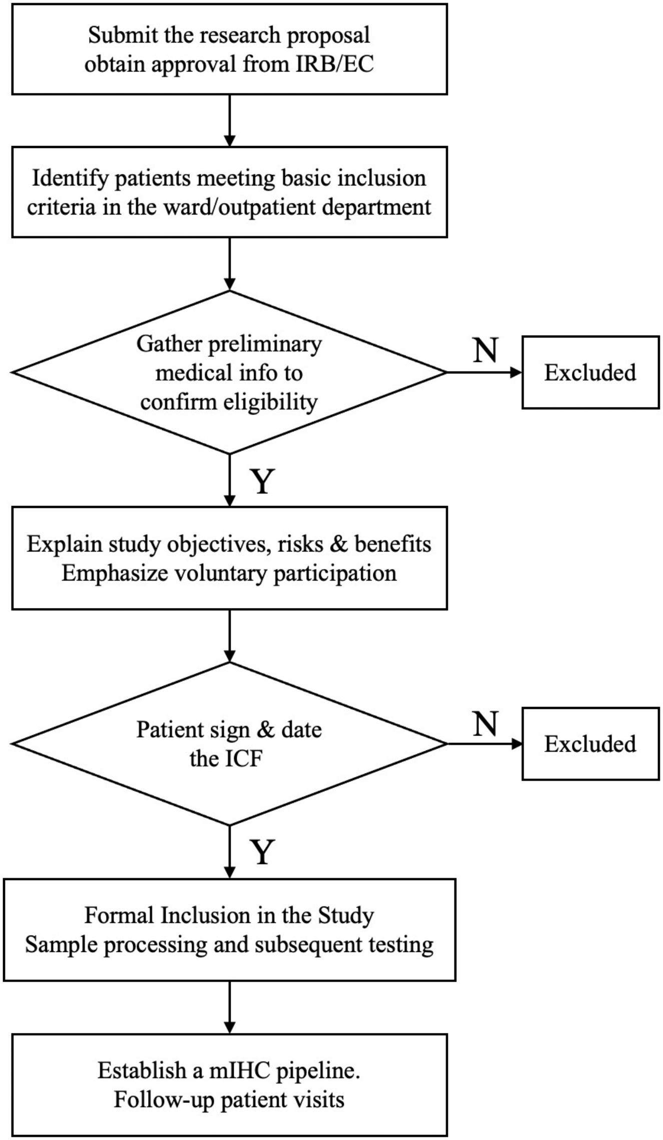

Supplementary Figure 2: Expression of ATRX in cells of glioma tissue.

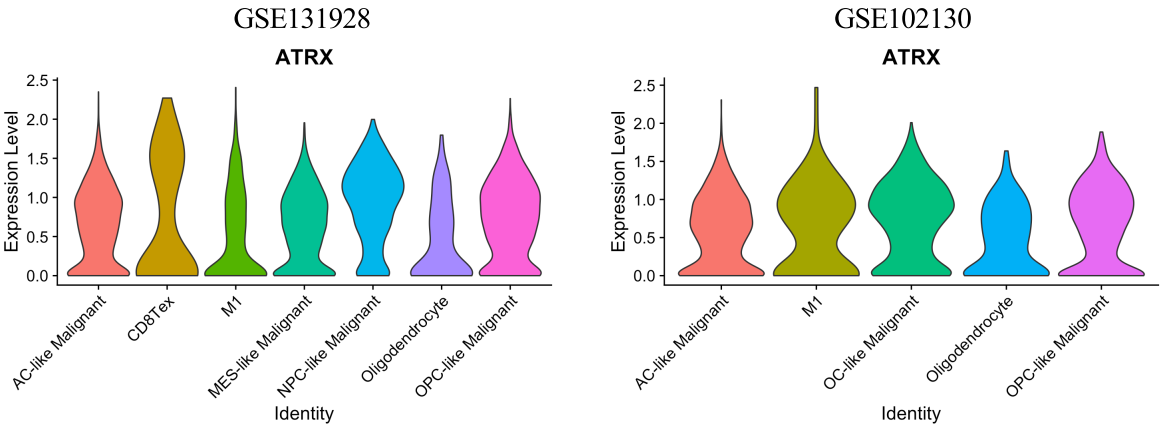

Supplementary Figure 3: ATRX mutation status' effect on prognosis in IDH mutant or wild-type gliomas.

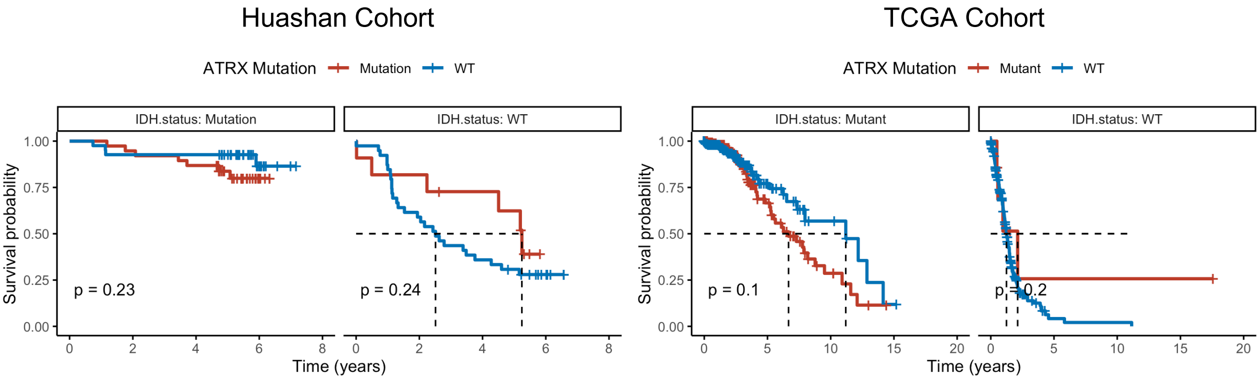

Supplementary Figure 4: Feature importance of the RSF model.

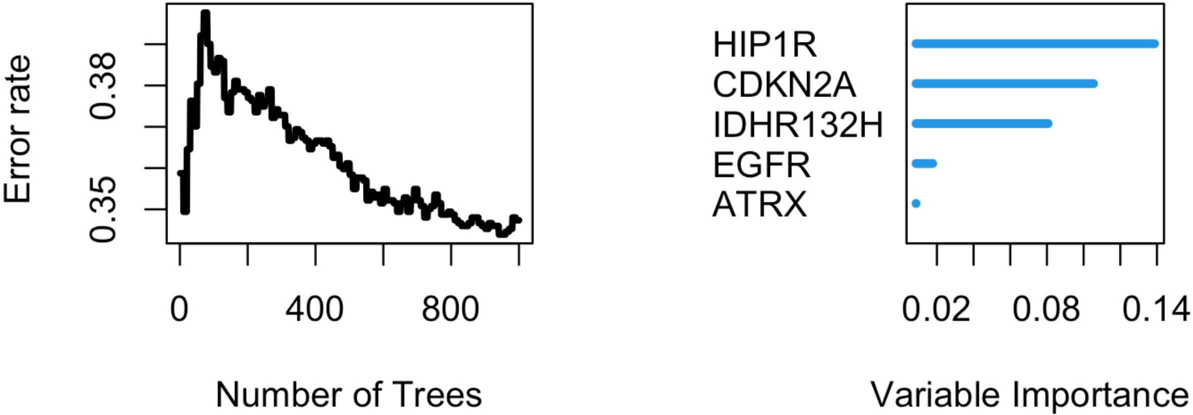

Supplementary Figure 5: Comparison of molecular characteristics between high-risk and low-risk glioma subtypes within IDH wild-type and IDH-mutant contexts.

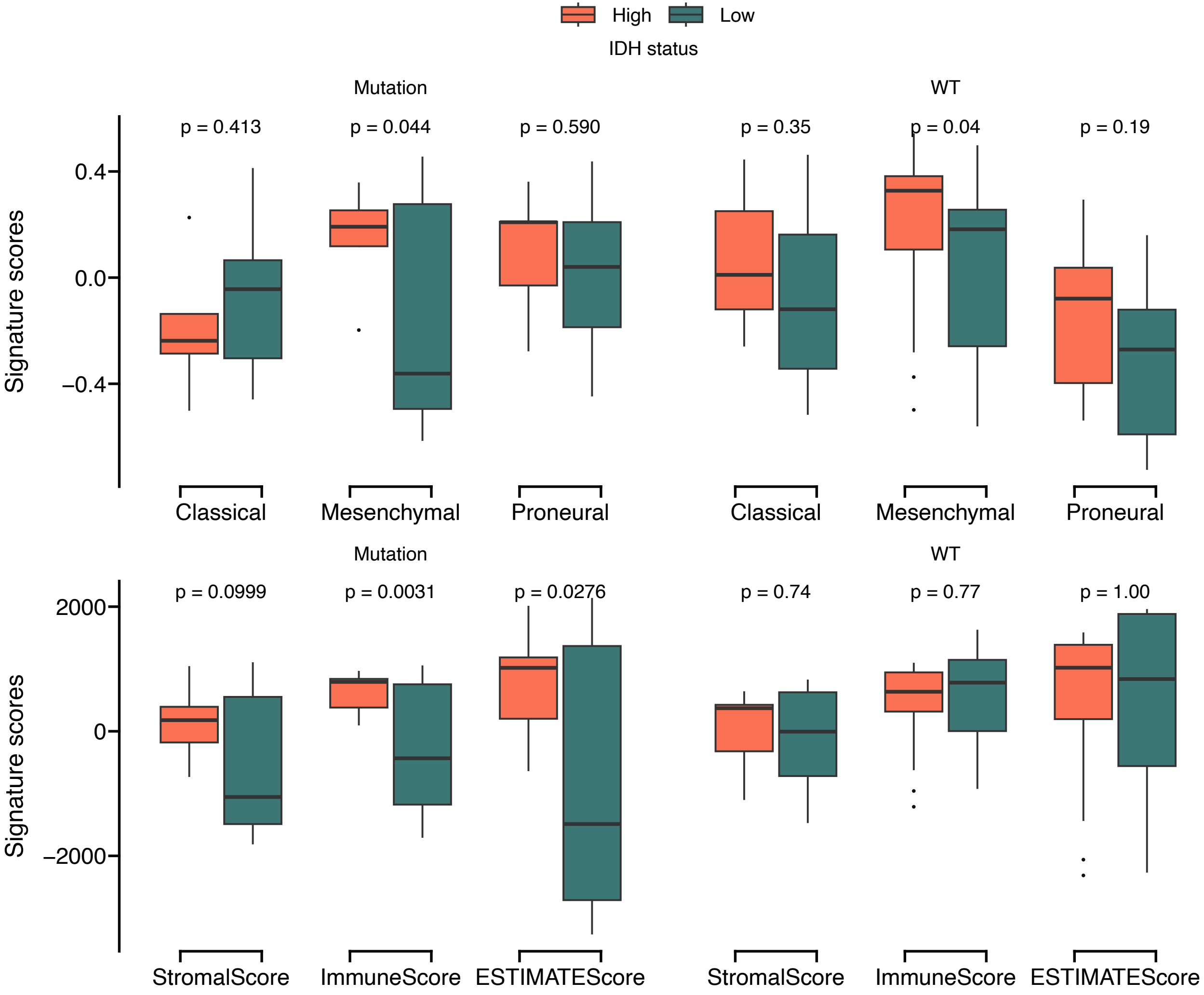

Supplementary Figure 6: Validation of mIHC-defined subgroups in TCGA-GBMLGG cohort.

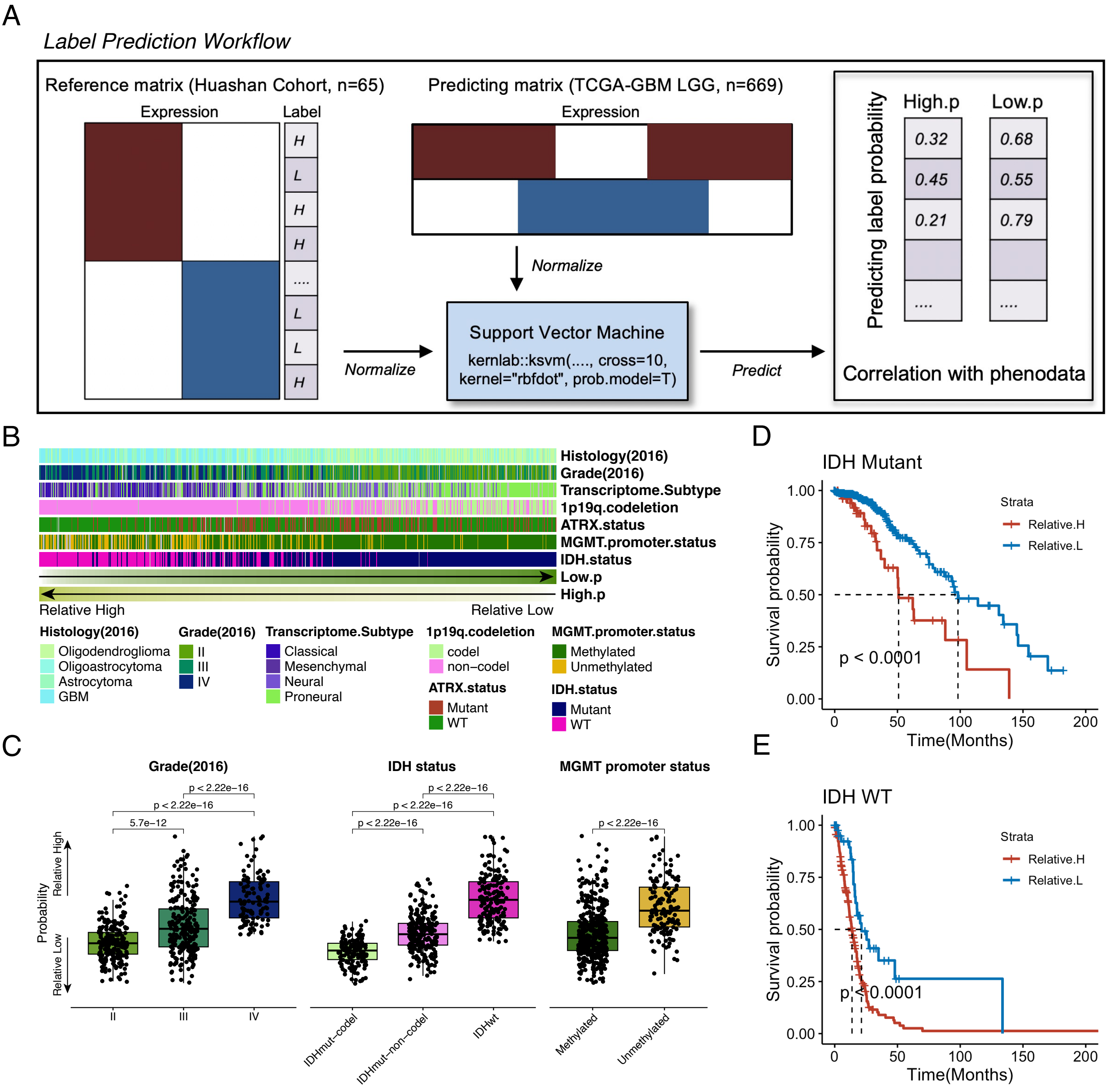

Supplementary Figure 7: Immune characteristics of SVM-based predicted subgroups of TCGA GBMLGG cohort.

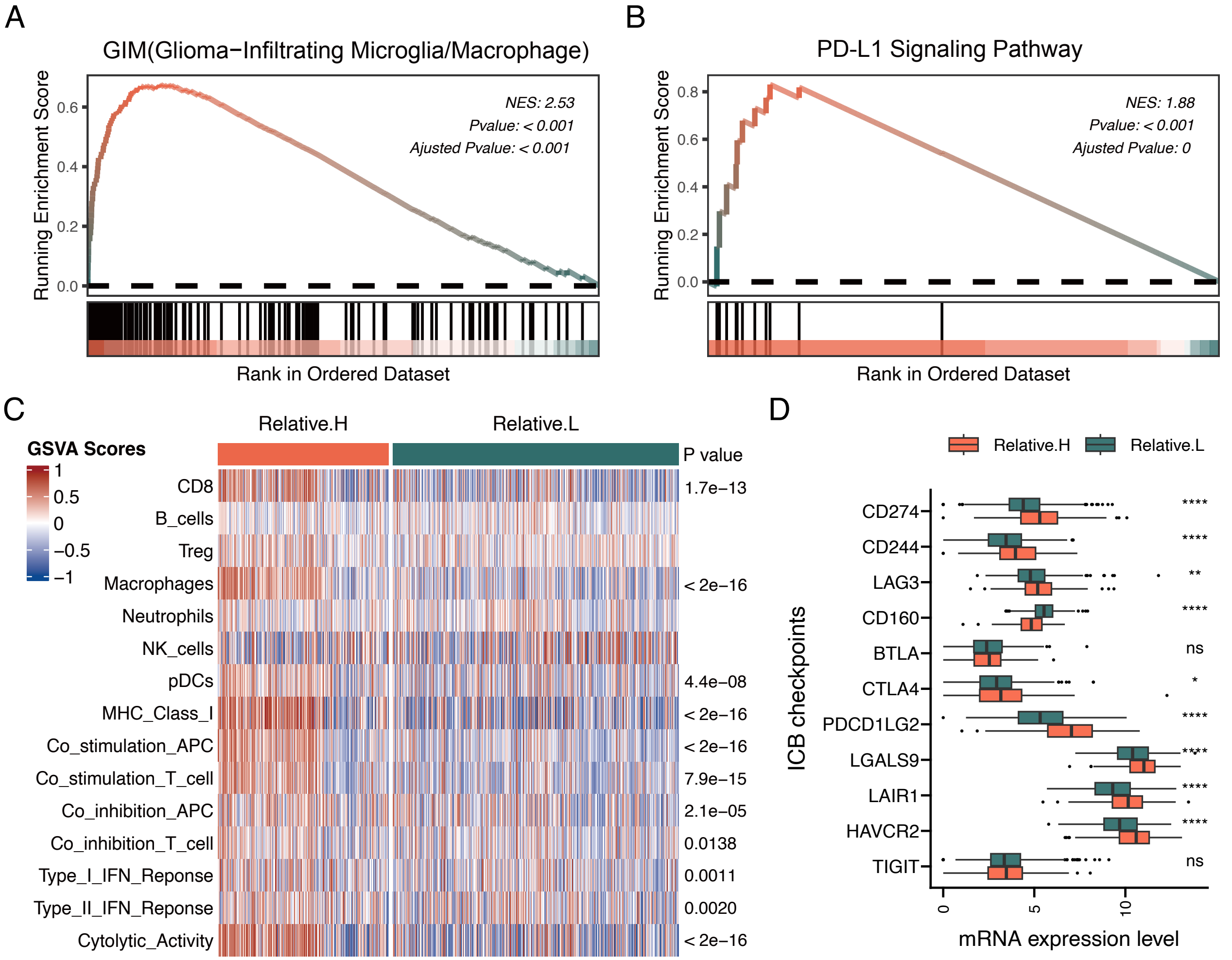

Supplement: Supplementary file 1 — Supporting Information [file MCO2-6-e70138-s001.pdf]
